# Psychosocial and environmental factors related to physical activity in middle-aged and older adults

**DOI:** 10.1038/s41598-023-35044-4

**Published:** 2023-05-13

**Authors:** Yi-Husan Lee, Sheng-Yu Fan

**Affiliations:** 1 Department of Nursing, National Cheng Kung University Hospital, College of Medicine, National Cheng Kung University, Tainan, Taiwan; 2 Institute of Gerontology, College of Medicine, National Cheng Kung University, No. 1 University Road, Tainan City 701, Taiwan

**Keywords:** Geriatrics, Human behaviour, Lifestyle modification

## Abstract

The social ecological model provides a comprehensive framework for understanding the multiple-level determinants of physical activity. This study explores the significant individual, social, and environmental variables and their interactions in relation to physical activity in middle-aged and older adults in Taiwan. A cross-sectional study design was implemented. Healthy middle-aged and older adults were recruited (n = 697) through face-to-face and online surveys. The data collected comprised self-efficacy, social support, neighbourhood environment, and demographic characteristics. Hierarchical regression was used for statistical analysis. Self-rated health (*B* = 74.74, *p* < .001; *B* = 101.45, *p* = .022) and self-efficacy (*B* = 17.93, *p* < .001; *B* = 14.95, *p* = .020) were the significant individual variables in both middle-aged and older adults. Neighbourhood environment (*B* = 6.90, *p* = .015) and the interaction between self-efficacy and neighbourhood environment (*B* = 1.56, *p* = .009) were significant in middle-aged adults. Self-efficacy was the most significant predictor for all participants, with the positive correlations of neighbourhood environment arising only for middle-aged adults with high self-efficacy. Policy making or project design should consider multilevel factors in order to facilitate their physical activity.

## Introduction

With the increase in size of the older population, maintaining and improving individual health are important issues. Studies have shown the benefits of physical activity for physical and psychological health^1–3^ and subjective well-being^4^. Older adults with low physical activity had higher likelihoods of sarcopenia^5^ and depression^6^. International and national organizations provide physical activity recommendation guidelines to promote physical activity cross the lifespan^7,8^.

The social ecological theory addresses the effects of multiple-level variables, including individual, social, and environmental variables, and the interactions cross levels in behaviour^9,10^. Individual variables are personal characteristics and beliefs^11^. One such belief is self-efficacy – the individual’s personal judgement about their own capability to produce a specific behaviour. Studies have shown self-efficacy to be positively related to physical activity in middle-aged adults (aged 35 to 64 years old)^12^, all adults^13,14^, and older adults^15–17^. Self-efficacy was a significant individual-level variable that related to physical activity across different ages^18^, and a review also showed that self-efficacy was the most consistently correlated with physical activity among the cognitive factors^19^. Physical activity has positive effects on heath, however, health status is also a foundation of taking physical activity^18,19^. Therefore, health status was collected as the covariate.

Social variables comprise interpersonal interactions and social support^11^. Older adults who receive social support from family members or friends engage in more physical activity^15–17,20,21^. Social connectedness is an important variable for older adults taking community-based group exercise^22^. Those attended senior club longer tended to have longer physical activity duration, and better quality of life^23^. However, a study has shown that social support is not significant for all adults^13^.

Environmental variables comprise the physical environment and facilities^11^. Physical activity in older adults has been linked to walkable access to amenities^24,25^, traffic conditions^26^, parks and facilities^15^, and the safety of the environment^17,25,26^. The results of a systematic review and meta-analysis supported the importance of the environment^27^. However, a study has shown that the neighbourhood environment is not significant because older adults tend to engage in physical activities at or near their homes^21^.

Research has focused on single aspects of predictors, and a small number of studies have explored the interactions among variables from different levels in older adults. For example, an interaction between self-efficacy and social support from friends has been identified. The effect of social support occurred in older adults with high self-efficacy^16^. However, this study did not consider the role of environment^16^. In addition, the positive effect of the neighbourhood environment on physical activity only occurred in those with high social support^20^ or those with high self-efficacy^28^. A study found older adults who had higher self-efficacy and perceived better social relationship and neighbourhood safety had higher physical activity^29^. However, these studies did not consider the interactions between the three levels of variables.

A few studies have included three levels of variables at the same time and have explored the interactions of different levels of variables. Older adults need physical activity to maintain or enhance health, however, the habit of physical activity may be developed from middle-age. In addition, there is uneven population distribution in Taiwan, and people have different environmental resources for physical activity. Therefore, this study included self-efficacy, social support, neighbourhood environment, and the interactions between different variables to explore the significant variables related to physical activity in middle-aged and older adults.

## Methods

### Study design

A cross-sectional survey using convenience sampling was conducted. Before the beginning of the study, ethical approval was obtained from an institutional review board of National Cheng Kung University Hospital (IRB number: A-ER-107–025).

### Setting

Two data collection strategies were used: a face-to-face survey and an online survey. The data collection period was from July 2018 to March 2019.The face-to-face survey was conducted in parks, community care centres, and a community activity centre of a special municipality in Southern Taiwan. These places were where older adults often engage in activities. Three trained research assistants with backgrounds in gerontology conducted the data collection. They had received training about the purposes, procedures, and contents of the measurements. An online survey link was posted on several social media sites. The purposes and contents of the measurements were also introduced clearly at the outset in the questionnaires. Therefore, the participants could complete the questionnaires by themselves.

The two collection strategies were used to increase sample size and diversity. For example, the face-to-face survey made it possible to recruit older adults and participants who did not know how to use the internet. The online survey made it possible to recruit participants from different areas, which increased the diversity of the neighbourhood environments. In the online survey, the aims and inclusion criteria were explained at the start of the questionnaire, and responses were excluded if the respondents did not fit the inclusion criteria, if there were inconsistencies in data, or if data were missing.

### Participants

The potential participants were healthy adults aged 45 years old and above and able to complete the standardised questionnaires. Those who had cognitive problems (e.g., dementia), severe physical diseases (e.g., heart disease or lung diseases), or dependence on others (e. g, severe disability and cannot take care of themselves) were excluded.

### Variables

The dependent variable was the amount of physical activity, and the predictors included demographic characteristics, self-rated health, self-efficacy of physical activity, and social support and neighbourhood environment for physical activity.

### Measurement

Demographic characteristics included sex, age, and educational level. Self-rated health status was also collected by asking ‘In general, would you say that your health is excellent = 4, good = 3, fair = 2, or poor = 1?’.

The Physical Activity Scale for the Elderly (PASE) was used to assess the level of the respondents’ physical activity in leisure time^30^. The scale includes six items relating to the preceding seven days: (1) sitting or sedentary behaviour, (2) walking outside the home, (3) light sport/recreational activities (e.g. fishing, billiards), (4) moderate sport/recreational activities (e.g. dancing, golf without a cart), (5) strenuous sport/recreational activities (e.g. aerobic dance, running), and (6) muscle strength/endurance exercises (e.g. hand weights, push-ups). The weights of the six activities were 0, 20, 21, 23, 23, and 30, respectively; and the weights were used to calculate total score of PASE^24^. The frequency (never = 0, seldom [1–2 days] = 1, sometimes [3–4 days] = 2, often [5–7 days] = 3) and hours per day of activity (less than 1 h = 1, 1–2 h = 2, 2–4 h = 3, and more than 4 h = 4) were rated. Total PASE scores were computed by multiplying the frequency of each activity (hours per day over a seven-day period) by the respective weights and summing all activities^27^. A higher score indicated a greater extent of physical activity in leisure time. The scale was translated into a Taiwanese format with good validity^31^.

Self-efficacy of physical activity was measured using a modified version of a previous scale^32^. It included seven items about the respondents’ confidence in their ability to engage in physical activity in specific situations, such as being in a bad physical condition, feeling depressed or in a negative mood, lacking time due to workload or household chores, lacking a place to exercise, lacking the skills needed, and bad weather. A 4-point Likert scale was used (4 = high confidence, 1 = no confidence). A higher score indicated higher self-efficacy in engaging in physical activities. The Cronbach’s alpha of this study was 0.85.

A 5-item scale was used to assess social support from family and friends for physical activity, such as engaging in physical activity with the participant or encouraging them to take physical activity^20,33^. A 4-point Likert scale was used (4 = strongly agree, 1 = strongly disagree). A higher score indicated greater social support for physical activity. The Cronbach’s alpha of this study was 0.74.

The physical activity neighbourhood environment scale (PANES) was modified^34^ including eight items: shops within easy walking distance of house, free or low-cost recreation facilities in the neighbourhood, sidewalks on most of the streets, visibility of people being physically active in the neighbourhood, facilities for cycling, transit stop within a 10–15 min walk from home, crime rate in the neighbourhood and safety of walking at night (reverse coding), and traffic on the streets making it difficult to walk (reverse coding). A 4-point Likert scale was used (4 = strongly agree, 1 = strongly disagree). A higher score indicated greater social support for physical activity. The Cronbach’s alpha of this study was 0.71.

### Study size

With regard to sample size, there were 12 predictive variables, and at least 144 participants were needed for each group (sample size > 12 * 8 + 50 = 144)^35^.

### Statistical methods

Descriptive statistics were used to present the characteristics of the participants. Hierarchical regression was used. The items sex, age, educational level, self-rated health, and the method of data collection (online versus face-to-face survey) were entered in Model 1, self-efficacy, social support, and neighbourhood environments were entered in Model 2, and interactions between predictors were entered in Model 3. The reasons of the three models were that the variables in Model 1 were controlled as covariates, and Model 2 could specifically test whether the variables about different levels predict physical activity significantly, and Model 3 could test whether the interactions had higher predictive ability than the variables.

Interactions comprised the product of two variables, with mean centring of two variables conducted prior to computing the product interaction term. For example, where the predictors were self-efficacy, neighbourhood environment, and interaction, [self-efficacy – mean of self-efficacy] × [neighbourhood environment – mean of neighbourhood environment]. The strategy of mean centring was used to avoid multicollinearity, and the variance inflation factor was used to test for multicollinearity. The significant moderating effect that the interaction between two variables are also presented in figures.

In addition to analysing all the participants, we analysed middle-aged adults (aged from 45 to 64 years old) and older adults (aged over 65 years old) separately. SPSS 21.0 version was used for the statistical analysis and a p value of less than 0.05 was taken as the significance level. The Strengthening of Reporting of Observational Studies in Epidemiology (STROBE) checklist was used for reporting this study^36^.

### Ethics approval and consent to participate

Ethical approval was obtained from the institutional review board of National Cheng Kung University Hospital (IRB number: A-ER-107-025). The participants who were recruited for the face-to-face survey signed informed consent forms. There were no illiterate adults involved in the study All procedures were performed in accordance with declaration of Helsinki' statement.

## Results

In the online survey, there were 626 adults who filled in the questionnaires, but 59 were excluded because they were not met the inclusion criteria and due to inconsistencies in data (the rejection rate was 9.42%). In the face-to-face survey, there were 138 adult participants, but eight were excluded because of missing data (the rejection rate was 5.78%). In the end, 697 participants were recruited, of which 514 (73.74%) were middle-aged adults. The mean of age was 59.47 years (SD = 8.74), 70.73% were female, and 69.54% had a college degree or a higher degree of education (Table 1). With regard to the source of participants, 567 (81.35%) were recruited from the online survey. The participants from the online survey were younger (*t* = -11.92, *p* < 0.001), had higher educational levels (*χ*^2^ = 221.75, *p* < 0.001), and had better self-rated health (*t* = 2.40, *p* = 0.017) than those recruited through the face-to-face survey.

**Table 1:** Demographic characteristics of the participants.

Variables	Total participants (n = 697)	Middle-aged adults (n = 514)	Older adults (n = 183)
Age	59.47 ± 8.74	55.36 ± 4.72	71.41 ± 6.45
Sex			
Male	207 (29.70%)	148 (28.79%)	59 (31.64%)
Female	490 (70.30%)	366 (71.21%)	124 (68.36%)
Educational level			
Elementary school or illiterate	58 (8.32%)	6 (1.17%)	52 (28.42%)
Junior high school	40 (5.75%)	22 (4.28%)	18 (9.84%)
Senior high school	115 (16.50%)	77 (14.98%)	38 (20.77%)
Colleges and above	484 (69.44%)	409 (79.57%)	75 (40.98%)
*Self-rated health*	2.51 ±0.68	2.53 ±0.70	2.47 ±0.62
*Physical activity*	354.85 ±314.67	332.19 ±304.24	413.41 ± 338.26
*Self-efficacy*	16.57 ±4.62	16.47 ± 4.65	16.82 ± 4.56
*Social support*	14.06 ±3.28	14.01 ±3.30	14.20 ± 3.28
*Neighbourhood environment*	24.44 ±4.42	24.57 ± 4.52	24.06 ± 4.11

The correlations between physical activity, self-efficacy, social support, and neighbourhood are presented in Table 2. For all the participants, demographic characteristics and self-rated health explained 9% of the variance in physical activity (*F* = 14.52, *p* < 0.001); self-efficacy, social support, and neighbourhood environment increased variance in physical activity by 9% (*F* = 25.17, *p* < 0.001); and the interactions between each of the two variables and three variables increased variance in physical activity by 2%. With regard to the significant predictors in Model 3, higher physical activity was significantly related to older age (*B* = 3.33, *p* = 0.011), higher self-rated health (*B* = 76.54, *p* < 0.001), and higher self-efficacy (*B* = 16.08, *p* < 0.001). In addition, there was a positive interaction between self-efficacy and neighbourhood environment (*B* = 1.70, *p* = 0.008) and negative interactions between self-efficacy and social support (*B* = -1.55, *p* = 0.012), and between social support and neighbourhood environment (*B* = -1.83, *p* = 0.012) (Table 3).

**Table 2:** The correlations between physical activity and other variables.

	Total participants					Middle-aged adults					Older adults				
	A	B	C	D	E	A	B	C	D	E	A	B	C	D	E
A. Physical activity	1					1					1				
B. Self-rated health	0.26**	1				0.27**	1				0.24**	1			
C. Self-efficacy	0.35**	0.26**	1			0.37**	0.28**	1			0.29**	0.18**	1		
D. Social support	0.15**	0.16**	0.35**	1		0.14**	0.17**	0.40**	1		0.15**	0.11	0.20*	1	
E. Neighbourhood environment	0.12**	0.18**	0.15**	0.20**	1	0.14**	0.14**	0.15**	0.16**	1	0.08	0.30**	0.12	0.33**	1

*p < .05, **p < .01.

**Table 3:** Results of hierarchical regressions.

	Total participants			Middle-aged adults			Older adults		
	Model 1	Model 2	Model 3	Model 1	Model 2	Model 3	Model 1	Model 2	Model 3
The method of data collection									
Face-to-face survey	REF	REF	REF	REF	REF	REF	REF	REF	REF
Online survey	1.36 (− 0.46, 1.95	1.10 (− 0.31, 1.46)	0.96 (− 0.05, 1.48)	0.98 (− 0.44, 1.60)	0.88 (− 0.55, 1.41)	0.79 (− 0.51, 1.44)	2.08 (− 0.85, 3.20)	2.00 (− 0.03, 2.69)	1.85 (− 0.21, 2.49)
Age	3.67** (1.66, 5.49)	3.43* (1.31, 5.60)	3.33* (1.26, 5.65)	5.03 (− 0.45, 10.30)	4.30 (− 0.94, 9.24)	4.28 (− 0.96, 9.18)	− 5.30 (− 16.81, 0.08)	− 3.07 (− 14.58, 2.82)	− 2.84 (− 13.82, 3.45)
Sex									
Female	REF	REF	REF	REF	REF	REF	REF	REF	REF
Male	39.67 (− 7.19, 91.34)	37.42 (− 7.46, 88.18)	32.36 (− 10.94, 84.23)	28.66 (− 25.13, 84.66)	29.36 (− 20.55, 84.28)	26.38 (− 24.81, 80.12)	66.43 (24.98, 152.16)	39.82 (− 26.22, 87.09)	28.47 (− 23.34, 80.16)
Educational level									
Junior high school and below	REF	REF	REF	REF	REF	REF	REF	REF	REF
Senior high school	12.96 (− 10.12, 42.69)	20.66 (− 22.40, 62.37)	25.11 (− 21.37, 61.59)	− 32.85 (− 145.74, 79.64)	− 36.47 (− 147.55, 84.66)	− 34.30 (− 143.80, 99.55)	16.35 (− 27.26, 66.01)	37.92 (− 81.70, 134.03)	32.54 (− 77.18, 123.49)
College and above	− 6.02 (− 15.12, 18.03)	− 14.66 (− 65.50, 95.34)	− 16.58 (− 66.25, 93.34)	− 45.29 (− 141.53, 74.59)	− 80.73 (− 177.63, 46.91)	− 75.25 (− 172.67, 52.09)	− 12.25 (− 8.11, 31.04)	29.81 (− 31.18, 94.71)	19.20 (− 26.23, 64.94)
Self-rated health	113.42*** (89.54, 115.13)	77.14*** (50.18, 116.15)	76.54*** (49.75, 115.39)	110.42*** (81.71, 153.01)	73.68*** (41.40, 112.06)	74.74*** (42.56, 112.99)	118.02** (53.27, 211.57)	99.21* (26.85, 196.22)	101.45* (25.20, 195.36)
Self-efficacy		17.32*** (12.75, 22.98)	16.08*** (11.58, 21.97)		19.32*** (13.79, 25.15)	17.93*** (12.71, 24.21)		16.38** (0.26, 24.22)	14.95* (1.36, 24.09)
Social support		0.63 (− 6.62, 7.31)	− 0.50 (− 7.78, 6.25)		− 2.03 (− 9.99, 5.61)	− 4.10 (− 12.10, 3.89)		8.02 (− 9.85, 22.06)	11.20 (− 7.00, 26.89)
Neighbourhood environment		5.42* (0.94, 11.16)	4.93 (− 0.62, 11.39)		6.95** (2.18, 12.99)	6.90* (1.87, 13.40)		− 3.49 (− 20.41, 12.34)	− 6.92 (− 19.01, 10.53)
Self-efficacy*Neighbourhood environment			1.70** (0.21, 2.86)			1.56** (0.46, 2.94)			1.85 (− 0.36, 2.18)
Self-efficacy*Social support			− 1.55* (− 3.37, − 0.46)			− 1.26 (− 2.73, 0.54)			− 3.48 (− 6.57, 0.31)
Social support*Neighbourhood environment			− 1.83* (− 2.99, − 0.75)			− 0.96 (− 0.44, 1.93)			− 3.27 (− 5.00, 0.34)
Self-efficacy*Neighbourhood environment*Social support			0.02 (− 0.27, 0.30)			0.03 (− 0.28, 0.38)			− 0.16 (− 0.86, 0.42)
R^2^	0.10	0.19	0.20	0.11	0.21	0.22	0.07	0.15	0.20
Adjusted R^2^	0.09	0.18	0.19	0.10	0.19	0.20	0.05	0.10	0.15
*F*	14.52***	18.21***	14.93***	10.02***	14.36***	10.88***	2.71*	3.41**	3.06**
R^2^ change		0.09	0.02		0.09	0.02		0.06	0.05
*F*		25.17***	5.22**		19.57***	2.47*		4.14**	2.47*

Model 1: age, sex, educational level, self-rated health, the methods of data collection.

Model 2: age, sex, educational level, self-rated health, the methods of data collection, self-efficacy, social support, neighbourhood environment.

Model 3: age, sex, educational level, self-rated health, the methods of data collection, self-efficacy, social support, neighbourhood environment, interaction between two and three variables.

*p < .05, **p < .01, ***p < .001..

**Figure 1:**
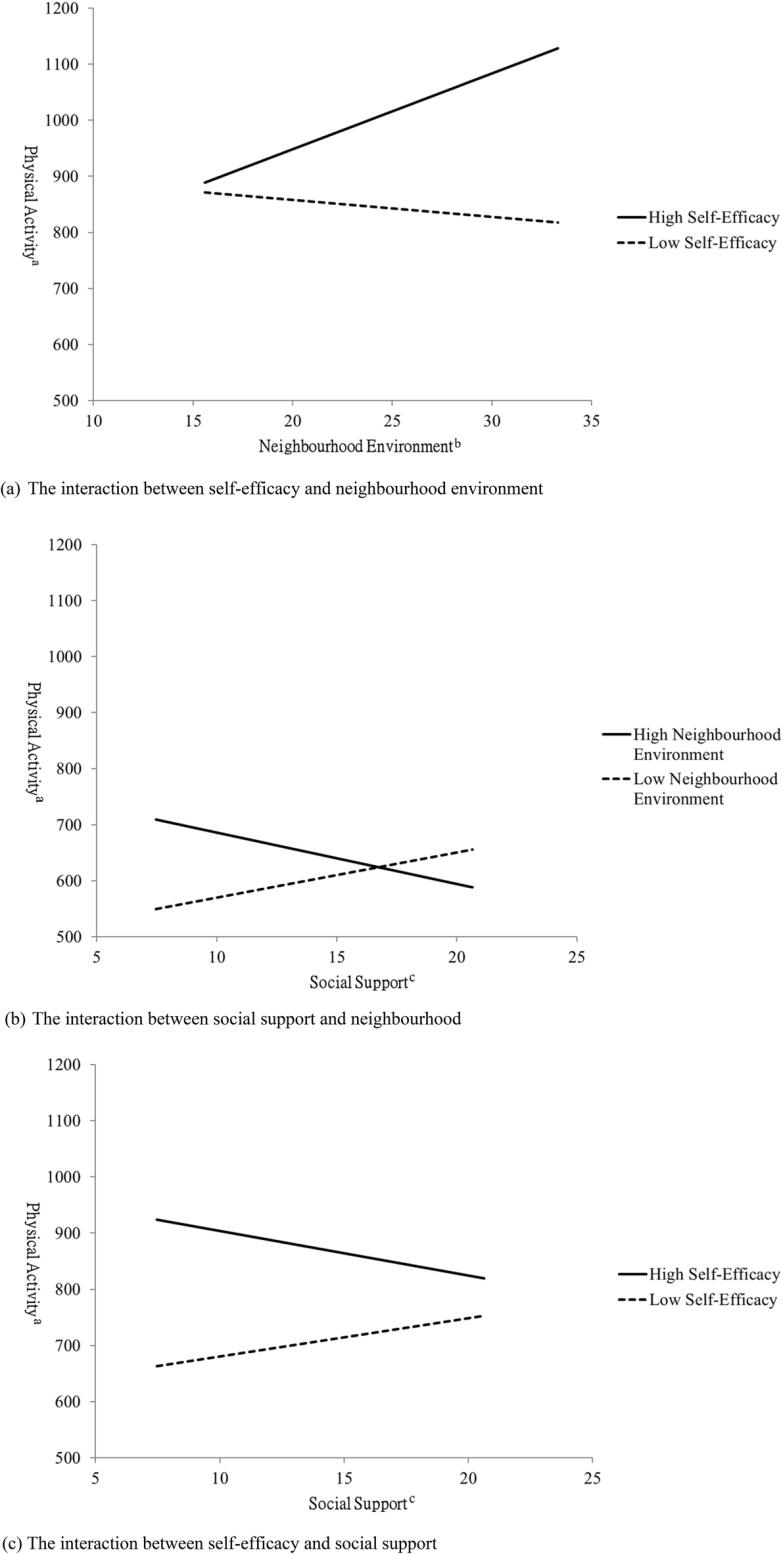
The interactions between variables of different levels for all participants (^a^The total score of PASE; ^b^The total score of neighbourhood environment scale; ^c^The total score of social support scale).

With regard to the interaction of self-efficacy and the neighbourhood environment, the slope of high self-efficacy was significant (slope = 13.42, *p* < 0.001). Participants with high self-efficacy had a higher level of physical activity when they had a better neighbourhood environment. The slope of low self-efficacy was not significant (slope = -3.00, *p* = 0 0.406) (Fig. 1a). With regard to the interaction between social support and the neighbourhood environment, the neighbourhood environment related to physical activity positively for participants with low social support (slope = 11.42, *p* = 0.002) but not for those with high social support (slope = -1.12, *p* = 0.744). For the participants with low social support, better neighbourhood environment related to higher physical activity (Fig. 1b). There was an interaction between self-efficacy and social support. With increasing social support, the participants with high self-efficacy had decreased physical activity (slope = -7.89, *p* = 0.126) whereas participants with low self-efficacy had increased physical activity (slope = 6.83, *p* = 0.115) (Fig. 1c).

For middle-aged adults, Model 1, 2, and 3 explained 11% (*F* = 10.02, *p* < 0.001), 21% (*F* = 14.36, *p* < 0.001), and 22% (*F* = 10.88, *p* < 0.001) of the variance in physical activity. The significant variables of physical activity in Model 3 included self-rated health (*B* = 74.74, *p* < 0.001), self-efficacy (*B* = 17.93, *p* < 0.001), neighbourhood environment (*B* = 6.90, *p* = 0.015), and the interaction between self-efficacy and neighbourhood environment (*B* = 1.56, *p* = 0.009). For older adults, Model 1, 2, and 3 explained 7% (*F* = 2.71, *p* = 0.033), 15% (*F* = 3.41, *p* = 0.008), and 20% (*F* = 3.06, *p* = 0.005) of the variance in physical activity. The significant variables in Model 3 were self-rated health (*B* = 101.45, *p* = 0.022) and self-efficacy (*B* = 14.95, *p* = 0.020) (See Table 3).

## Discussion

This study used a standardised quantitative methodology to explore the potential effects of individual, social, and environmental variables on physical activity in middle-aged and older adults. Self-rated health and self-efficacy were the significant variables in both middle-aged and older adults. In addition, neighbourhood environment and the interaction between self-efficacy and the neighbourhood environment were significant variables in middle-aged adults.

As previous studies, both middle-aged and older adults with better self-rated health^12,13,18,19^ tended to have more physical activity. There was a dual process in which health was the foundation of physical activity, and then physical activity enabled improved health^2,37^. Older adults need functional fitness to take physical activity.

In addition, the results showed the significant role of self-efficacy in physical activity in middle-aged and older adults, which supported the findings of previous studies^12–17^. As Pan et al.^13^ reported, self-efficacy had a greater effect than social support and neighbourhood environment in middle-aged and older adults. Belief about personal ability and the confidence to take physical activity and overcome barriers were crucial for behavioural action.

Walkability, convenience, and safety of the neighbourhood have been shown to have positive effects on physical activity^15,17,24–27^, the effect mainly being for middle-aged adults. Most of the participants in this study lived in urban areas, where had better infrastructure for physical activity. Those lived in rural areas might not have environmental resources for physical activity. The effect of neighbourhood environment was significant in Model 2 of total participants, but not significant in Model 3, because the interactions between variables were more significant. As in the case of previous studies^28,38^, the finding of this study also showed the interaction between self-efficacy and the neighbourhood environment. The neighbourhood environment was associated with physical activity only among participants with high self-efficacy. Middle-aged adults with low self-efficacy may not take more physical activity, even with a better neighbourhood environment.

In contrast to previous studies^15–17,20,21^, this study and that by Pan et al.^13^ showed that social support was not significant for physical activity when controlling individual and environmental variables. Self-efficacy and the neighbourhood environment had a greater influence on physical activity than social support did. Research showed that that social support could improve physical activity in those with high self-efficacy^16^, whereas the results of this study revealed that an increase in social support was positively related to physical activity among those with low self-efficacy. On the other hand, there was interaction between social support and the neighbourhood environment^20^. Social support is associated with physical activity only among people who have a poor neighbourhood environment. When social support is low, a good neighbourhood environment is a resource for physical activity; but when social support is high, the neighbourhood environment is not related to physical activity. Perhaps self-efficacy has a greater influence than social support does, and those with high self-efficacy are able to take the initiative with regard to physical activity. Thus, social support only helped those with low self-efficacy and a bad neighbourhood environment.

For all the participants, age was a positive predictor – participants who were older had more physical activity. A potential explanation is that older adults have more leisure time and more health concerns than middle-aged adults^39^, and might spend more time in physical activity. On the contrary, health should also be considered at the same time. Older adults with health problems or severe diseases may have less physical activity.

Regarding clinical implication, self-efficacy is the most significant variable and strategies focused on self-efficacy can be applied, such as organizing physical activities, providing role models and feedback, and building up confidence for both middle-aged and older adults. The accessibility of physical activity facilities and a walkable and safe environment can make it easier for middle-aged adults to engage in physical activity. Furthermore, social support can be provided to those with low self-efficacy or who live in areas inconvenient for physical activity. Policy makers can address health education about self-efficacy, provide accessible environment for physical activity, as well as promote formal or informal interpersonal relationships for physical activity.

Some limitations to this study should be acknowledged. First, the participants had good health, and the results cannot be generalized to middle-aged and older adults with severe health problems. Second, there was imbalance in the sample, as 25.62% of the participants were older adults and PASE was designed for older adults. The regression analyses were conducted in all participants and then in different age subgroups. The reason was to provide more information about the relationships between physical activity and different levels of predictors in different age groups. However, these two groups lack comparability, and for example, we could only infer that self-efficacy related to physical activity in both groups, but we could not verify whether self-efficacy had great power in middle-age adults than older adults. Third, the extent of physical activity and the condition of the neighbourhood environment were rated on the basis of the participants’ memory and self-perception. Fourth, the convenience sampling method was used and some demographic, socioeconomic, and geographic variables related to physical activity were not included, including per capita income, occupation, number of children in the family, and urban or rural areas, which then caused the low explanatory power of the models.

Regarding future studies, stratified sampling combined with the random method may be used to recruit the participants, some socioeconomic variables related to physical activity could be collected, and wearable devices and geographic information systems could be employed in future studies to collect objective data. Furthermore, interventions involved multi-levels variables can be conducted, e.g., promotion of self-efficacy, social club of physical activity, or modification of neighbourhood environment, to test the roles and effects of different variables on different ages.

In conclusion, self-rated health and self-efficacy were the most significant variables of physical activity in middle-aged and older adults. For middle-aged adults, the neighbourhood environment and the interaction of self-efficacy and the neighbourhood environment were also significant.

## Data Availability

The data that support the findings of this study are available on request from the corresponding author. The data are not publicly available due to ethical restrictions.

## Abbreviation

PASE: The Physical Activity Scale for the Elderly
